# The Impact of Smartphone Apps Designed to Reduce Food Waste on Improving Healthy Eating, Financial Expenses and Personal Food Waste: Crossover Pilot Intervention Trial Studying Students’ User Experiences

**DOI:** 10.2196/38520

**Published:** 2022-09-02

**Authors:** Therese Fostervold Mathisen, Frode Ramstad Johansen

**Affiliations:** 1 Faculty of Health, Welfare and Organisation Østfold University College Halden Norway; 2 Faculty of Computer Sciences, Engineering and Economics Østfold University College Halden Norway

**Keywords:** smartphone app, food waste, healthy eating, diet, automatic, registration, global sustainability, financial expenses

## Abstract

**Background:**

Global sustainability and individual health need coordinated attention. While individuals are recommended a healthy diet to reduce the burden of noncommunicable diseases, global attention to natural resource conservation is also needed. The latter specifically means effective measures to reduce food waste.

**Objective:**

This pilot study evaluates the experiences of students and effect from using smartphone apps designed to reduce food waste on personal healthy eating, financial expenses, and food waste.

**Methods:**

A total of 6 students from different study programs (mean age 24.7, SD 2.9) were recruited to evaluate 2 different apps designed to reduce food waste and to register food consumption, food waste, and financial food expenses before and after the app trials. The apps evaluated were the commercially available TotalCtrl Home and Too-Good-To-Go. Results were analyzed by mixed methods, comprising statistical analyses for quantifiable data and thematic analyses for qualitative data. The apps were used separately in random order, each for 1 month. Primary outcome was user expectations to and experiences from the use of the apps, which were obtained by semistructured interviews. Secondary outcomes were changes in food waste volume, financial food expenses, and healthy eating. While information on food waste and food expenses was obtained by weighing food waste and registering food costs for 2 weeks before and after app trials, scores for consuming healthy diets were calculated from registered food records by scoring criteria matched to national recommendations for healthy eating.

**Results:**

Awareness on food waste increased after app trials, but experiences with apps pointed toward several potential for technical and content improvements. The students reported that there were too many manual operations in the apps to induce permanent use (TotalCtrl Home), that services seemed more concerned about the producers’ interests than the individual’s needs (Too-Good-To-Go), and that they missed a composite app that included functions to promote healthy eating and overview of budget and expenses as well as of food waste (both apps). Use of apps designed to reduce food waste and personal costs and to improve healthy eating did not result in any measurable effects, that is, no change in food waste (mean change 0.81, SD 1.5 kg; *P*=.13), healthy eating (mean change –0.24, SD 0.43; *P*=.24), or personal food expenses (mean change 47.5 NOK or US $4.8, SD 416.9 NOK or US $42.5; *P*=.39).

**Conclusions:**

Apps may aid in increased awareness of food waste at the producer and consumer levels. Large-scale studies with longer duration are needed to see if apps may induce measurable changes in food waste, healthy eating, and financial expenses.

## Introduction

Food waste means the loss of produced food meant for consumption by humans, and is estimated to encompass about 20%-30% of all produced food [1-3]. The loss of edible food means loss of important resources, such as energy and capital, which puts a constant stress on natural resources (involves all stages of the product life cycle, from production to destruction), including the production of greenhouse gases that cause global warming [4-7]. To counteract these severe consequences, effective measures to reduce food waste are requested, and the European Parliament has put forth a goal to reduce food waste by 50% within 2030 [7].

A significant amount of food waste occurs at the individual consumer level, with an estimated loss of 179 kg per capita per year in the European Union [8-10]. As such, motivating the individual for making personal efforts in reducing food waste is essential. Importantly, study findings suggest that individuals are prone to commit to food waste–reducing behavior if they are educated about the consequences (acting to reduce feeling of guilt), and if they feel expectation and joint commitment from the society [11]. On the contrary, if they have a busy time schedule and experience the food waste–reduction behavior to require extra efforts, they are less likely to engage [11,12]. Younger generations may be less knowledgeable about reuse of leftover foods, and as such engage in higher food waste behavior than older generations who are typically more educated on food management from their experience of periods with shortness of food supply or limited personal finances [13,14]. Nevertheless, young adults are highly aware of the environmental consequences from our consumer culture in general, and as such may be motivated to change behavior if making an impact. If efforts concurrently favor their own economic situation, organized systems easing the efforts to reduce food waste could enhance their motivation to engage [15].

Today, young adults are massive users of digital technology, with over 95% in the advanced economies being smartphone users [16]. Smartphone apps have become convenient programs for many daily activities, and are perfect channels to deliver personalized and socially responsible interventions [17]. Previous research on apps for health or behavioral interventions among young adults reported that the participants did not find the apps personally relevant, but revealed tentative willingness to test them [18]. Hence, it is important that the apps have features that appeal to the young consumer. A recent structured review of available apps to promote sustainable waste management behaviors identified the 6 most persuasive strategies applied with the aim of influencing the users’ behavior [17]. The first prominent features listed were a reduction of complex app tasks to make the use of the app easier and a personalized content. Next, an experience of a real-world belongingness by bringing information about the app company and surface credibility (ie, competent look and use) were listed as typical. Lastly, functions such as reminders and self-monitoring were provided in an attempt to motivate and engage the user. Self-monitoring may be specifically reinforcing when payoff is measurable and real, and not only considerations for the environment, but also personal profits could embody such achievements. For young adults such as students, financial restraints often limit their leisure spending and participation. If education and awareness about the impact of slight modification of everyday behavior can make them realize financial savings, they may improve motivation to engage in such a behavior.

Importantly, the elements typically employed in apps for behavioral changes relate to the 3 factors highlighted as necessary to assist behavioral change: motivation, ability, and triggers [17,19]. As such, apps may emerge as promising aids for the individual to comply with the intention to reduce food waste and concurrently experience personal rewards such as saving money. With such intentions in mind, the health of the individual must also be taken into consideration. Individuals in the modern society need to improve their diets to expect healthy life [20,21]; hence, the consideration for the environment (ie, reduce food waste) and motivation for financial savings must include attention to the personal diet. The intention of this pilot study was to explore whether smartphone apps can help students to reduce their personal food waste, improve their habitual intake of a healthy and varied diet, and reduce financial expenses on food.

## Methods

### Design and Procedure

**Figure 1:**
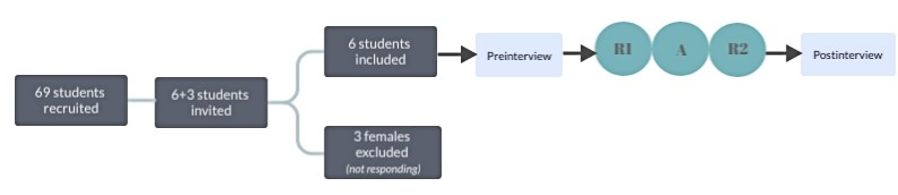
Study flow. Three females did not respond to invitation, and as such three more females from the recruited pool were invited. In total 6 students (3 males and 3 females) consented to participate. Note: Preinterview, individual interviews on expectations. R1, 2-week preregistration period on food waste, diet and dietary expenses. A, two-month period trying two digital phone applications consecutively. R2, 2-week postregistration period. Postinterview, individual interviews on experiences.

This is a pilot intervention study exploring students’ expectations to and experiences from using smartphone apps that aim at reducing food waste and evaluating its effects on food waste, healthy eating, and financial food expenses. As such, this study combines efficacy and usability testing, and provides a broader perspective and evaluation as previously highlighted in a viewpoint paper [22]. The study included a preperiod with baseline registrations and interviews on expectations, a trial period in which 2 different apps were explored for 1 month each by all the participants in a random crossover order, and a final postperiod repeating the baseline registrations and interviews on experiences (Figure 1).

### Participants

We recruited students from a university in south east of Norway during the winter of 2020/2021. Recruitment messages were distributed through the university’s internal communication system (Canvas), asking for students interested in digital interventions to reduce food waste, and rewarding the 3 months’ participation with a financial compensation of 7500 NOK (US $765). In total, 69 students responded, from which a total of 9 were invited, and 6 were finally included (Figure 1). Students were sorted by sex, housing condition, study, and experiences with food waste reduction before selected invitations were sent (from the pool of recruited participants) to present a group with balanced characteristics.

### Outcomes

This pilot study followed a mixed methods design, including quantifiable and qualitative outcome data.

All participants were instructed to manually register their food consumption, food expenses, and financial expenses for food in the 2 weeks before and after the app trial period. The registration was by use of a precoded MS Excel (Microsoft, Inc.) document. The information and instructions on registrations and use of registration tools (ie, kitchen scale and excel document) were provided in a synchronous digital meeting, and any expenses related to the procurement of the kitchen scale were covered by the project sponsor. Food consumption was registered by noting details on time for consumption, type and volume of food consumed, and finally noting any leftovers not consumed (added to the total registration of foods wasted from the private household). Foods wasted were noted separately in the MS Excel document after weighing foods before they were thrown in the litter. All expenses for food, including receipts from shopping, were registered in the same MS Excel document on a separate sheet. Foods consumed were evaluated according to a healthy index, foods wasted were evaluated according to weighed volume, and food expenses were compared before and after the app trial solely by calculation comparisons.

The healthy food index created for this study was kept concealed from participants to avoid biases, such as changes occurring simply from the awareness of being evaluated. The index consisted of a total score ranging from 0 to 5, and was calculated on the following premises: 1 point if more than half of the daily grain/grain products consumed were whole grain products; 0.5 points for each portion of the “5-a-day” recommendation for fruits and vegetables (each portion being 100 g, and providing a maximum of 2.5 points); 0.5 points if sugary drinks were absent/nonsugary drinks were preferred; and 1 point for consuming fish. These criteria match the international and national recommendations for a healthy diet, highlighting the health benefits of increasing the intake of plant-based foods and fiber, reducing intake of sugar, and by replacing fish for meat [20,23].

Participants were interviewed following a semistructured manual once before the trial, focusing on previous experiences on app use to reduce food waste, increase healthy eating, or control economy, and on expectations of the intervention period. Finally, the participants were interviewed after the intervention about their experiences from using apps to reduce food waste and increase healthy eating, as well as to understand how these affected their financial expenses. See Multimedia Appendix 1 for interview guides. Both authors studied the transcribed interviews (in Norwegian) and approved the English translation of quotes. Translation of quotes was done with the intention to reflect the oral spoken language by the participants (ie, not primarily considering the optimal grammatical form of expression/written language).

### Intervention: Smartphone Apps

The 2 apps used in this trial were Too-Good-To-Go (TGTG) and TotalCtrl Home. Each app was designed to aid in reducing food waste and to provide the user with a chance for reduced food expenses.

TGTG covers major European cities and is an app designed to help restaurants and stores with unsold food surplus to reach potential customers. As such, the stores may be able to reduce food waste and also have a small income from the surplus sales. The stores may post their food surplus in the app with a reduced price and with a defined time frame in which customers may bid for and collect the items. The products are reserved by the customer when TGTG confirms their order with a reservation confirmation. The customer has the possibility to filter the results in the app in consideration of the availability of products to reserve, of the pickup hour, or of the nature of products contained in “magic bags.” The pickup time will normally be in a period of 10-30 minutes but can be both shorter and longer. The profit for the customer is obvious, a potential to reduce personal food spending by having access to price-reduced foods and by aiding in reducing global food waste.

TotalCtrl Home is an app designed to aid in food waste reduction at the individual level, to inspire cooking and as such to facilitate better financial utilization of food investments. The functions are about providing an overview of the food content in the home, to offer recipes based on the foods registered in the personal app “kitchen” and specifically for those food items registered with an expiry date, and to assist with the creation of a shopping list based on suggested menus included in the app register. New food items procured for the household may be registered in the app by scanning the barcode or by manually typing in the details, resulting in an addition to the app kitchen. The foods may be registered as stored in the freezer, the refrigerator, or the cabinets, and must be manually updated if changes occur (ie, used for cooking, changing storing location, wasted).

### Statistical Analysis

The quantitative data were analyzed using SPSS Statistics version 27 (IBM, Inc.). The healthy food index (total score), amount of food wasted (in grams), and personal financial expenses were analyzed for any changes from pre- to posttrial. With regard to the small group of participants, and the robustness of the Student *t* test, such parametric analyses were applied to explore the pre-post changes.

The interviews were analyzed thematically as suggested by Braun and Clarke [24]: transcribing and familiarizing with the data, generating initial codes for the data, identifying and reviewing themes by collated codes, naming themes, and producing the report. During the final stage, rich extracts were chosen for illustration of themes, and finally, the analyses were checked according to the original research question.

### Ethics Approval

This trial and its data collection and storage plan have been approved by the Norwegian Agency for Shared Services in Education and Research on April 3, 2021 (id number 832471). All included participants signed an informed consent before participation in this trial.

## Results

### Overview

The group of 6 recruited students had a mean age of 24.7 (SD 2.9) years and represented 6 different study programs (nursing, information technology, teaching, digital media and design, data science, and economy and administration), and were equally distributed with regard to gender. All participants lived in a single-person housing unit, and reported to exercise a mean number of 2.7 (SD 1.5) sessions per week. As many as 5 of the 6 participants reported to have previously tried some initiatives to reduce food waste, and 5 participants reported to have tried apps to register and analyze their diet.

### Part 1: Expectations About Participating in a Project Exploring the Effect of Apps Aiming to Reduce Food Waste, Contribute to a Healthier Diet, and Save Money on Foods

#### Personal Needs Before Global Needs

The students were first asked about their motivation for attending this project. Most students indicated the need to reduce personal expenses for food as the most important motive to engage in reduction of food waste. One student talked about her routine of always turning to the desk with last-minute offers in the grocery store, because there often are affordable offers. Having her friend doing the same thing kind of normalized that behavior to her. The promise of saving money by using an app and the recommendations from relatives and friends about all the cheap food made available through an app were important motives reported by most students. But few had continued to use the apps, because of less positive experiences.

It’s mainly about the economy. How much can you save by reducing personal food waste, or...Well, for some, saving money is more motivating than reducing food waste due to environmental issues.

Male student, 25 years

...you get a lot of bakery goods, and I know I’m not going to be eating all those wheat buns. And then...I don’t know, well, they end up in the freezer and then you eat them some other time you’d fancy some bakery goods. But this is the main reason I do not use those apps any more. They sort of..., well, yes, you do save a lot of money on those eight wheat buns, but you weren’t going to buy eight wheat buns in the first place.

Female student, 22 years

Other than the experiences of being offered bakery goods for snacks rather than foods for main meals, the short time slots and competition to be in reach of the offers were experienced as a large limitation to engage further. The experiences from being primarily offered snacks further draw attention to the personal health perspective when joining such app initiatives. One student told about her initial positive experiences on using apps to reduce food waste, as this made her save a lot of money. But as her awareness on personal health and reasonable body weight regulation increased, she started looking for other apps.

Everything starts from myself, like what I want first. I just want...I don’t like my body to feel out of control, so that’s why I try to control myself, and to be controlling it, I need some tools.

Female student, 27 years

This female student was very aware of the global issue with food shortages, and was raised with a view of food as something to praise. The student really wanted to explore new foods, but yet determined not to produce unnecessary waste caused by personal pickiness. One way to accommodate these contradictions was to use an app where food stores could offer cheaper last-minute foods. The student first found this as a nice way to try new foods, while concurrently helping the stores reduce food waste and have income for these last-minute offers. However, as the student increased her awareness of healthy foods, the first positive experiences vanished with several disappointments (ie, foods being trashed because they were not suitable for the student’s needs).

#### Global Awareness Challenges Personal Pleasure

When the students were asked what they thought could be a meaningful personal contribution to reduce food waste, they all responded with suggestions related to personal cooking and shopping behavior. They mentioned that they made sure leftovers from cooking and meals were reused and not thrown away in the litter. They argued to plan the meals, to know about and wisely use what is present in the refrigerator, and to save any leftover from meals for later use. Attention to what is bought for the household, by knowing what is already in the refrigerator and what is actually needed, was another suggestion made by most students. One student reflected over her awareness of food waste from the personal household:

Well, I live alone, which means that all I put into my refrigerator must be consumed by me...or at least I must in some practical way make sure it is consumed. And then you have these fantastic, green recyclable bin-bags; where you see exactly the volume of food you trash.

Female student, 22 years

One student also argued that moderation would be an important move to reduce food waste in a global perspective—simply not to overconsume food. The overconsumption by single individuals does not only challenge personal health, but also the global availability of natural resources. Implicit in the reflections by these students is also a premise of purchasing only what you as a single individual or personal household would need and what you know you would be able to consume. Referring to the latter, one student reflected on this, finding the global consideration to be a personal limitation:

I also want to try a new food, a new type of food; just for the experience. And maybe it just might taste better. But it may sometimes cost me more. Because, you know, if I try a new pie, and then if I don't like it, then probably I'm going...not going to eat it.

Female student, 27 years

#### Artificial Intelligence, and Not Too Detailed, Please

All but 2 had already tried different types of apps to reduce food waste, or apps to increase insight into personal diet and health. While the former was primarily motivated by potential personal economic savings, the latter was by different reasons. Still, while the health motives varied from sport performance, body weight control, to illness control, they all agreed that detailed nutritional information was not in their interest, or could induce issues with food:

No, I mean...I think it brings too much stress. I cannot bear to be that detailed. Really, I want to live a normal, balanced life.

Male student, 25 years

One student had also wanted to find an app to gain control over her personal financial expenses. Here too, details, advanced analyses and functions were not needed, specifically if these additions meant charges for use. As such, she chose to use a simple MS Excel program to log her incomes and expenses. Her system, including logs of economy and logs of nutritional intake, with the aim to keep low food waste, resulted in the use of multiple systems that needed to be operated manually.

That’s when I searched for the applications that could help me to gain control, and control is not only about health or finance, but also my lifestyle and my time. Because if I do something manually, it will be really time consuming. Like, it’s automatic. I don’t have to type in things; that save time and is more convenient.

Female student, 27 years

The idea that apps should be intelligent and demand less work and manual operations was mentioned by most students. Further, an app should not be too advanced with too many functions or information, but rather provide an easy overview of financial expenses, energy intake, or food waste. Such overviews could be comparisons on a weekly or monthly basis, to see if one keeps track of low food waste or financial savings. Both the perspective of time use and the burden experienced by manually operating the apps were mentioned. Just as important was the preparatory work one has to do to provide information to the app, such as if one has to weigh the food consumed for dietary calculations, or the food *not* consumed for food waste calculations. Several mentioned the idea of taking pictures of items, or scanning a barcode, and that this should provide the app with enough information to do the necessary registration and calculations. Additionally, an app that could aid as an extended source of memory and creativity was held as desirable. It should remember what you already have at home when you are in the grocery store, should suggest menus based on your food stock, and should automatically calculate the nutritional content in what you cook and consume.

### Part 2: Experiences From Using Apps to Reduce Food Waste, Improve Diet, and Gain Financial Control

#### Main Experience

After a period of 2 months, none of the students felt the apps had helped them in reducing their food waste noticeably. However, most felt their *awareness* of food waste had improved, and that there had been an economic impact from better planning (eg, knowing about your food stock before going to the grocery store, avoiding impulsive food shopping, and by reusing leftover foods).

#### Big Brother

The awareness of food waste, both at the individual level and at the wholesaler and producer levels, was reported to be brought into everyday attention simply by joining this project. But the functions provided by the apps were also reported as effective tools. The experience from viewing repeated offers in the TGTG app increased the understanding of the volume of food wasted at the producer and wholesaler levels. Also, the registration of foods in the TotalCtrl app augmented the attention of food in personal stock.

I feel that I have a larger responsibility in reducing food waste after being made aware of how much food is wasted from the different wholesalers. I could easily be waiting a few more minutes at the store after picking up my pre-booked bag if I notice there are more bags to be saved from being wasted. ( - ) It’s actually ok to realize that I can have this responsibility; I mean, if its only about me eating crisp bread rather than my usual bread, I’m more than willing to do that.

Female student, 22 years, on experiences from TGTG

This project has made me more aware of due dates. I look for due dates maybe once per week in my fridge; are there any items I need to plan to use, in order to consume them before the due dates.

Female student, 23 years, on experiences from TotalCtrl

The students further mentioned the pre- and postregistration period to be eye-openers not only to personal food waste, but also to the economic potential and personal health.

It could be incidental, but it is likely that when you register your diet, and the registrations say pizza and burgers for four days in a row... (–) You become more aware of it, when you see it like that, written in words. You end up thinking; this is not good for you.

Male student, 29 years, on experiences from pre- and postfood registration

Nevertheless, the scenario of being recruited to a study and feeling monitored may cause a Hawthorne effect, in which the participants are very aware of their behavior and of the intention of the study. As such, it may be difficult to say if the efforts made (eg, reducing food waste) are an effect from app use or from being observed and monitored.

It’s a bit like...when you know you are being observed, then you want to perform, and as such I think I have consumed healthier food. (–) But I think the app is there for the better. It will be kept in my phone. I definitely will...continue to re-use food leftovers in other meals. I have learned new things during this period.

Female student, 23 years, on experiences from TotalCtrl

And as far as I understood that during the period we used the app, I actually had much better control over the fridge and everything that was there.

Male student, 22 years, on experiences from TotalCtrl

Considering that most students wanted to continue using the apps after the study, it seems logical to say that the apps are helpful tools. This means that consumers first need to be recruited for app use to realize the potential within them, and then to find routines to continue using them during hectic periods rather than to fall back to old behaviors.

It’s mostly about personal motivation. That...kind of, the busier you are, the more likely such new things fade out. If you have short time and need to cook dinner quickly, then you’ll just reach for something simple on your way home. You just forget about it in a hectic everyday life.

Male student, 22 years

#### Food Waste: The Consumer or The Producer Perspective

One of the main objectives reported by the students about joining a study aiming to reduce food waste was to experience economic benefits. While a student said she had noticed that her receipts were getting shorter by using the apps, few said they had experienced any favorable economic effect. Most reported first to be fascinated by the cheap offers made with the TGTG, but to experience increased personal food waste and uneconomical spending in the long run. To be effective at reducing food waste, students argued the apps had to facilitate choices from several wholesalers in the living area and not only kiosks and bakeries, to not be confined to limited pickup times, and to possess a personal willingness to eat what is offered rather than according to a planned diet or personal likings.

I'm very picky when it comes to food that is due date or approaching expiration date, and a bit picky with food in general, and if you never know what you're getting in a bag like that then...much is thrown in the litter anyway.

Male student, 29 years, on experiences from TGTG

Sometimes I cannot consume the foods because of my diet. So, I have to give it away. But if I cannot find someone to give it away to, I have to throw it away. So, the apps sometimes also increased my food waste.

Female student, 27 years, on experiences from TGTG

Its definitely cheap, but I feel like I’m...wasting money when I do not want to eat what I get.

Male student, 25 years, on experiences from TGTG

Most students argued that the TGTG moved the issue with food waste from the wholesale to the consumer level, as the offers were mostly about bakery goods in larger amounts than a person would repeatedly be willing to buy and eat. As such, most had discontinued the use of TGTG other than on occasions where they actually wanted some sweets.

I don’t know...it may not be according to the idea with the concept, but rather than giving away magic bags [bags with unknown content to the consumer], they could list the items available online. The consumers can then pick...four items in a bag to an affordable price, rather than having 10 items in a bag and end up throwing half of them.

Male student, 29 years, on experiences from TGTG

But while most found the TGTG concept more concerned about the producers’ interests and was neither an affordable economic solution nor promoting more healthy foods or less personal food waste, 1 student had many positive experiences and personal solutions to what others identified as problems. Rather than becoming annoyed by the large volume of bakery goods offered, she made sure the leftovers were stored in the freezer or given to friends. Furthermore, she had gained an experienced knowledge on what wholesalers to engage with, to find affordable offers with decent foods. She saved money on foods by engaging with the TGTG, and that it gave her opportunities to also have fresh food, like the bread of the day, by being willing to wait until the end of the day. She had continued using the TGTG based on the positive experiences, not only due to the feeling of really helping in reducing local food waste, but also motivated by personal interests.

Previously I ate very much according to routines and habits: like I always ate the same for breakfast. But now, when I suddenly end up with fish pudding in my bag, and I never used to have this previously, I come thinking; “Oh, ok, so this will be what I eat today”. So...I don’t know, but this makes me feel I actually eat more varied now.

Female student, 22 years, on experiences from TGTG

Compared with the TGTG app, the TotalCtrl app also included other functions, and provided the participants with an increased awareness of foods traits. Although they found the manual registration unnecessary, inconvenient, and time consuming, this step alerted them regarding expiry dates of foods and motivated them to better utilize foods within the household. The digital overview of the food stock and the provision of recipes within the app inspired some to buy less by impulse, to plan for optimal utilization of foods within the food stock, to keep an eye on expiry dates of the food stock, and to do more cooking. As such, providing accountability increased the potential for reducing food waste.

You come to think about it more often. I haven’t reflected on it in a similar way previously, although I’ve been good at utilizing food I have available. But now, I more consciously think “I can use this left-over in other meals/menus too”.

Female student, 23 years, on experiences from TotalCtrl

I feel like I've done more cooking in a way. When you get the recipes, and get some inspiration from it, you feel like doing it.

Female student, 23 years, on experiences from TotalCtrl

The students talked positively about the functionality in the TotalCtrl app, providing an overview of the food stock, the inspiration provided by menu suggestions, and the ease in transferring recipes to a shopping list. But this ironically also meant more cues for additional food shopping, which would be in contrast to reducing food waste.

If I have milk in surplus that is due date, I’ve tried to find recipes based on milk. This makes me realize a need for other ingredients which I need to buy at the grocery store, and if I’m first going to the grocery store, then I could in reality buy something else and that I would not need the milk at all.

Female student, 22 years, on experiences from TotalCtrl

#### Version 1.0

The experiences of the students in using the 2 different apps point to the many beneficial effects on awareness, accountability, positive experiences of reward by saving money or attaining cheap food, and personal contribution to reduce global food waste.

It was really nice, with many recipes and related stuff. And that you can, with a simple click, just add all the ingredients from a recipe to your shopping list, it was really smart.

Male student, 29 years, on experiences from TotalCtrl

I’ve experienced some eye openers. I have not thought of these thing previously....like if a thing is due date. (-) Now, living alone, I need to keep an eye on these things, and this project has contributed to increased awareness. Yes, now I check due dates maybe once per week, so that I’m able to make plans.

Female student, 23 years, on experiences from TotalCtrl

However, several shortcomings and a potential for improvements of the apps were identified. The lack of consumer focus by the TGTG, mainly about reducing food waste at the producer level, was mentioned by most. The TotalCtrl, by contrast, was better suited for the consumer needs and included more functions. But one clear limitation was the lack of prize information, and as such, no function could help consumers in getting control over the budget or expenses. Furthermore, a small database and a slow development and update process were mentioned. The function with the barcode scanning first brought excitement, being automatic and quick, but later caused frustrations. These experiences also resulted in some ideas for improvement.

The app is so...manually. When I tried to scan an item, the database was not big enough and it did not recognize the item, so therefor I had to type it in again, and then I also had to type in about expiration date, and sometimes you pick up an orange, and the...the item don’t have any given expiration date, right?

Female student, 27 years, on experiences from TotalCtrl

It did not give me any good overview. I think it took too much time to register (manually) in the app-fridge. And then I had to make sure I updated it....like if I ate something, then I had to update that it’s no longer in the fridge. Just like that – it just became too much work, by something that should be very simple.

Female student, 22 years, on experiences from TotalCtrl

I think maybe what could have made the function [barcode scanning] even better, would be that the scans I make of new items, like a box of macaroni, will automatically enter a database. Then when the next person scans this same barcode, there will appear notice that I know something about it. Yes, so everyone in a way helps to expand the data base with different items.

Male student, 29 years, on experiences from TotalCtrl

If you have registered what you have in the fridge and in the freezer, and if you find a recipe in the app, then it automatically knows that you have four items from the recipe in the fridge, so that it does not add them to the shopping list, for example.

Male student, 29 years, on experiences from TotalCtrl

There are still some challenges that are difficult to resolve when it comes to the app initiatives for reducing personal food waste. Potentially this can be solved by adding a reminder function that notifies if food items have not been in use after being placed in the food stock.

I thought the app would be smart and estimate time left for an item for me, but it did not. Like, I bought one kilo of avocados and left them in the fridge, and I did not remember about them, and they got rotten. So, I don’t think its really helpful in the case of reducing food waste.

Female student, 27 years, on experiences from TotalCtrl

### Effects From Use of Digital Apps

**Figure 2:**
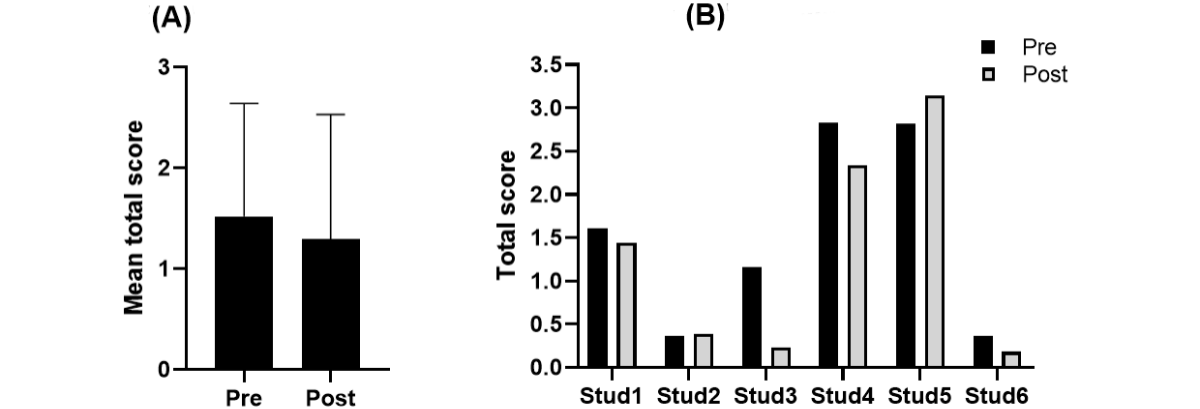
The healthy diet index (HDI). Panel A illustrates the group mean (StD) total score in HDI (ranging from 0-5, with higher scoring indicating more healthy diets) before (pre) and after (post) trials with the two digital apps; and panel B illustrates the individual changes in HDI per student. Stud: student.

There was no statistically significant change in the healthy diet index score in the group, with a mean change of –0.24 (SD 0.43; *P*=.24) from pre- to posttrial (Figure 2A). The individual changes point toward some different personal experiences (Figure 2B), but overall with small effects. There were few students consuming fish, at least on a regular basis, and among the 3 students reporting regular intake of fruits and vegetables, portions of 1-3 per day were most typical. While 2 participants were concerned about doing their own cooking, the sample in general was characterized by high consumption of take-away foods.

**Figure 3:**
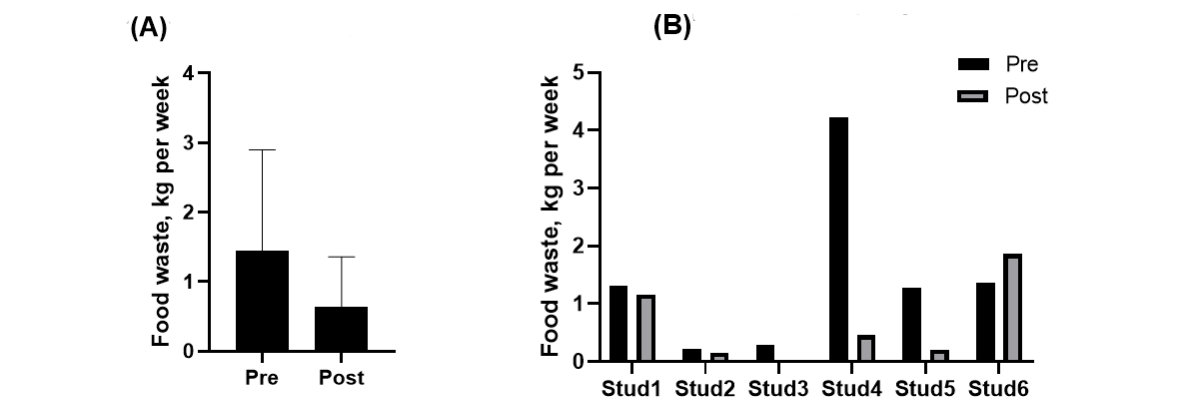
The mean food waste before and after app-trials. Panel A illustrates the group mean (StD) total food waste before (pre) and after (post) trials with the two digital apps; and panel B illustrates the individual changes per student. Stud: student.

There was no significant reduction in food waste in the whole group of students (Figure 3A): mean change 0.81 (SD 1.5) kg (*P*=.13). One student specifically experienced a large positive effect on food waste reduction during the trial, but the remaining students revealed negligible effects (Figure 3B).

**Figure 4:**
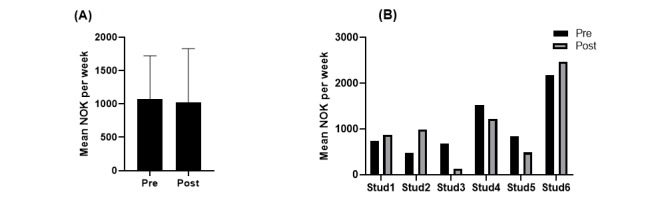
The mean economic expenses for food before and after app-trials. Panel A illustrates the group mean (StD) total expenses before (pre) and after (post) trials with the two digital apps; and panel B illustrates the individual changes per student. Stud: student.

There were no statistically significant differences in food expenses from before to after the app trials (Figure 4A): mean change 47.5 (SD 416.9) NOK or US $4.8 (SD US $42.5; *P*=.39). The lack of change was seen as a result from half increasing their spending and half decreasing their spending (Figure 4B).

## Discussion

### Principal Findings

This pilot intervention study evaluated the experiences of using smartphone apps designed to reduce food waste and financial food spending, and whether such apps could result in measurable changes, including an improved diet quality, among students. The main findings were that the apps increased awareness on food waste, but that they neither reduced food waste or financial expenses on food, nor improved healthy eating. Experiences from using the apps point toward a need to increase automatic operations and reminder functions, and increased attention to the needs of the personal consumer.

As much as students are aware of global climate issues, and truly engage in improving these, the immediate need to safeguard personal health and financial situation seems to overshadow the commitment to the environment. This does not imply an ignorance of the global perspective, rather they need to cope with their own situation before finding the capacity to focus on a broader perspective. With apps promoted to embrace both perspectives, there is a low barrier to engage. However, as long as the apps are more concerned about the producers’ interests than the consumers’ interests, the engagement and interest from the consumer may be short lived.

The affordable offers in these less-food-waste apps may actually conquer the objective of gaining control over personal health, which may be seen as a consequence when apps are primarily designed to match the interests of the producers and wholesalers. Young adults, such as students, are in situations that may cause more awareness about the relationship between behavior and health. This may simply be trying to live on their own for the first time, establishing a personal lifestyle behavior, or because moving to new places means large changes in (food) culture. If there is an increased awareness on how foods impact our health, commercial initiatives on reducing food waste may actually be counterproductive, specifically as long as the apps are predominantly designed for the producers’ interests.

One important finding from this study is what emerges as the main determinant for efforts in reducing food waste, whether it is on the personal level or the producer’s level. Although concerns on the environmental impact of food waste are real, the motivation and willingness to engage depend on the experienced *economic* outcomes. While the apps are designed from the producer’s perspective, that is, reducing food waste in store by offering last-minute offers, thus ensuring some income for the producer, the food waste in a wider perspective is not reduced. The customer may first be motivated by the initiative from these apps; users can contribute to less food waste while having edibles at a reduced price. However, when the edibles are foods they originally did not want or need, they realize that personal money is spent on unnecessary expenses and food waste is simply replaced from the producer level to the consumer level. Less food waste does not seem to be realized based on pure idealism, but must include an individual profit impacting individual resources if the parts are to engage.

### Comparison With Prior Work

Our findings, pointing to a need for personal reward, have previously been highlighted as important for successful behavioral changes and from app use specifically [17]. The experience of personal reward, being financial or other forms of profit (eg, attaining goods or improving health) after making personal efforts, is specifically reinforcing for the motivation to engage in a specific behavior. Self-monitoring is regarded as one of the most effective measures for behavioral change, as it brings alertness on effects from personal behavior, thereby enhancing commitment [17,25]. The feeling of reward is easily attainable when the efforts have been registered and measured, and such apps may aid in performing self-monitoring.

The students in this study had different experiences from the 2 apps in the trials, but most agreed that manual work and complex tasks reduced their interest to engage with the app. The lack of long-term compliance in the use of apps has previously been highlighted, and too much manual work was mentioned as one important limitation [26]. Some also mentioned the desire for more personalized content, like an opportunity to register specific dietary considerations to have more personalized offers and content in the apps. Besides the preference for automatic functions and personalized content, there was the need for reminders (eg, for the content in food stock getting close to the expiry date). These findings confirm previous literature reviews on the necessary functions of apps [17]. The apps did increase the awareness on global food waste, self-monitoring of food consumption, food waste registration, insight into the potential for health improvements, economical savings, and personal responsibility for reducing food waste. This points to the potential for many personal and global beneficial outcomes, although the interests for engagement with such apps first need to be triggered. Underlining previous findings, education about consequences of wasting food and experiencing a joint commitment from the society may be important triggers [11]. But as previously reported, busy periods, such as those during examinations, experienced by students mean less motivation to engage, if reducing food waste means extra personal efforts (eg, manual work in the apps, needing to visit the stores at specific times to have the affordable offers, or cooking) [11,12].

### Limitations

The strengths of this pilot study are the use of weighed food and waste records before and after the trials, and the precise registrations of expenses by registration of all receipts. Further ensuring the credibility and generalization of the current findings is the inclusion of students of both sexes, from different study programs, and with different experiences in food registration and food waste engagement. The study also contributes with data on the content, usability, and efficacy of commercial apps, thus informing professionals in making more targeting apps [22].

Limiting the generalizability of our findings are the small group of participants and the use of only 2 apps. Nevertheless, referring to a previous review of available apps designed to reduce food waste, only 11 were identified, and findings on user satisfaction were in line with those currently reported [17]. Importantly, the financial reward that the students received from participating in this study may have shaped the outcome of this study; rewards such as financial savings are important in motivating individuals to achieve reduction in food waste. Still, financial initiatives are commonly used as recruitment strategy in studies, and students are specifically concerned about their restricted economy, which naturally results in the preoccupation of or priority of saving money. Finally, of specific importance relating to the lack of quantifiable effects from this trial is the fact that students were facing their examination period during the posttrial measurements. Hence, most students said they did not have time to engage with app offers and recommendations, or do cooking or think of economical savings, and as such ate much take-away and on-the-go foods.

### Conclusion

Our findings suggest that apps designed to reduce food waste must combine the personal interests by the consumer (ie, economy, health, not too complex or detailed functions, and less manual operations) with the interest of the producers/wholesaler (economy). A frequent update on foods within the app database, inclusion of new providers, and maintenance and development of operating functions are important efforts from the app providers. Embracing these needs and perspective may better contribute to a total, global enhanced utilization of edible resources, and as such reduces food waste. Further prospective studies need to be conducted, preferably with different population samples, to examine whether apps designed to reduce food waste truly do have any beneficial effects.

## Appendix

Multimedia Appendix 1 Supplementary data; interview guide referred to in the method-paragraph.

## Abbreviations

TGTG: Too-Good-To-Go
