# Supplementary material for: The Impact of Smartphone Apps Designed to Reduce Food Waste on Improving Healthy Eating, Financial Expenses and Personal Food Waste: Crossover Pilot Intervention Trial Studying Students’ User Experiences
Source: JMIR Form Res. 2022 Sep 2;6(9):e38520. doi: 10.2196/38520 (PMC9482070; doi:10.2196/38520)
Supplement: Multimedia Appendix 1 [file formative_v6i9e38520_app1.pdf]

Appendix

Interview guidance on *expectations* to the intervention.

What attitudes and experiences does students at the University have to digitalization of diet and food waste?

I. How can you as a single consumer contribute to reduce food waste?
II. Have you ever used any app or similar to reduce food waste?
    a. If so, what are your experiences with these?
    b. What do you think is important functions to make you want to use such apps?
III. Have you ever tried to achieve any control and insight of your own diet, in order to improve economy or health?
    a. If so, what have been the apps/technology used?
IV. Have you used any apps or similar of personal diet to achieve insight of nutritional intake?
    a. If so, what are your experiences?
    b. What do you think is important functions to make you want to use such apps?

Interview guidance on *experiences* from using apps designed to aid in reducing food waste.

Do use of digital tools for diet and food waste result in more varied and healthy diet, and a reduction of food waste? If so, why?

I. You have now used two different apps who are designed to reduce food waste, improve diet, and/or providing an overview of financial expenses on food. How did you experience these? Do you have any specific thoughts on one/any of these?
II. Did you experience to waste less food? Did any of these contribute to make you be more aware of consuming the foods in stock and/or left-overs from meals?
III. Did you experience any increased awareness of food waste with these apps? Did you have any eye-openers?
IV. Did you experience that these apps provided you with a better overview or control of personal economy and/or food expenses?
V. Did you experience to eat healthier by using these apps? What do you specifically mean by eating healthier?
